# Increase in susceptibility to insecticides with aging of wild *Anopheles gambiae* mosquitoes from Côte d’Ivoire

**DOI:** 10.1186/1471-2334-12-214

**Published:** 2012-09-13

**Authors:** Mouhamadou S Chouaibou, Joseph Chabi, Georgina V Bingham, Tessa B Knox, Louis N’Dri, Nestor B Kesse, Bassirou Bonfoh, Helen V Pates Jamet

**Affiliations:** 1 Centre Suisse de Recherche Scientifique en Cote d’Ivoire (CSRS), Abidjan, 01BP1303, Côte d’Ivoire; 2 Vestergaard Frandsen, 13 Aborlebu Crescent North Labone, P. O. Box KA 30201, KIA, Accra, Ghana; 3 Vestergaard Frandsen, Chemin de Messidor 5-7, Lausanne, CH - 1006, Switzerland; 4 Vestergaard Frandsen, Waiyaki Way, ABC Place, PO Box 66889–00800, Nairobi, Kenya

**Keywords:** *Anopheles gambiae* age, Insecticide resistance, Vector control

## Abstract

**Background:**

Appropriate monitoring of vector insecticide susceptibility is required to provide the rationale for optimal insecticide selection in vector control programs.

**Methods:**

In order to assess the influence of mosquito age on susceptibility to various insecticides, field-collected larvae of *An. gambiae s.l.* from Tiassalé were reared to adults. Females aged 1, 2, 3, 5 and 10 days were exposed to 5 insecticides (deltamethrin, permethrin, DDT, malathion and propoxur) using WHO susceptibility test kits. Outcome measures included the LT_50_ (exposure time required to achieve 50% knockdown), the RR (resistance ratio, i.e. a calculation of how much more resistant the wild population is compared with a standard susceptible strain) and the mortality rate following 1 hour exposure, for each insecticide and each mosquito age group.

**Results:**

There was a positive correlation between the rate of knockdown and mortality for all the age groups and for all insecticides tested. For deltamethrin, the RR_50_ was highest for 2 day old and lowest for 10 day old individuals. Overall, mortality was lowest for 2 and 3 day old individuals and significantly higher for 10 day old individuals (P < 0.05). With permethrin, the RR_50_ was highest for 1 to 3 day old individuals and lowest for 10 day old individuals and mortality was lowest for 1 to 3 day old individuals, intermediate for 5 day old and highest for 10 day old individuals. DDT did not display any knockdown effect and mortality was low for all mosquito age groups (<7%). With malathion, the RR_50_ was low (1.54 - 2.77) and mortality was high (>93%) for all age groups. With propoxur, no knockdown effect was observed for 1, 2 and 3 day old individuals and a very low level of mortality was observed (< 4%), which was significantly higher for 5 and 10 day old individuals (30%, P < 0.01).

**Conclusion:**

Results indicate that for *An. gambiae s.l.* adults derived from wild-collected larvae, there was an influence of age on insecticide susceptibility status, with younger individuals (1 to 3 days old) more resistant than older mosquitoes. This indicates that the use of 1 – 2 day old mosquitoes in susceptibility assays as recommended by the WHO should facilitate detection of resistance at the stage where the highest rate of the resistance phenotype is present.

## Background

Insecticide-based interventions remain the principal vector control measure in malaria endemic countries. Insect proteins targeted by insecticides generally become insensitive to these compounds through point mutations such as the *kdr* mutation, altered acetylcholinesterase and GABA mutations [1-3]. Metabolic resistance involves biochemical transformation of an insecticide through mechanisms including enhanced detoxification or sequestration, ultimately reducing the capacity of the insecticide to interact with a target insect protein and cause mortality. Many studies have described the primary involvement of esterases, cytochrome P450 monooxygenases and glutathione-S-transferases in conferring resistance [4,5]. Major progress has been made in the last decade in identifying genes associated with insecticide resistance and structural changes affecting their functions [1-3]. However, knowledge on vector insecticide resistance status, changing trends of resistance in target vectors and their operational implications remain basic requirements to guide insecticide use in disease control programmes. This knowledge can provide a basis for selecting insecticides and for ascertaining continued susceptibility to insecticides already in use. Standardization of test methods for monitoring insecticide resistance is important as it ensures comparability of data from different sources, for different test populations and assessment periods. Standardized test include the World Health Organization (WHO) susceptibility test [6]. There are three general factors that can affect outcomes from WHO susceptibility test: (i) the physiological status of mosquitoes used in assays, i.e., whether adult females are unfed, blood fed, semi-gravid or gravid; (ii) the age of the adult mosquitoes used; and (iii) the temperature/ humidity under which insecticide exposure is conducted. The WHO guidelines [6] for performing assays advise the use of non-blood fed adult females at 24–48 hours post-emergence, within a test environment of 23-27°C and 70 – 80% relative humidity. However, in many field studies, the age of mosquitoes used varies widely [7-9]. Reduced phenotypic resistance in older mosquitoes has been observed in several laboratory studies [10,11], but very few studies have explored this phenomenon in natural populations.

Since outcomes from resistance tests can influence decisions in insecticide-based intervention programs, we investigated the extent that different ages of wild population of *An. gambiae* adult mosquitoes can influence phenotypic resistance to insecticides of four different classes used in malaria control, and whether the current methodology is likely to result in variable classification of resistance status.

## Methods

*Anopheles* pre-imaginal stages (L1 to L4 instars) were collected via ladles within rice farms from the village of Tiassalé in Côte d’Ivoire in April 2010, a time of year corresponding to the transition from the dry to the rainy season. Tiassalé is located 110 km northwest of Abidjan and is surrounded by irrigated rice fields. Rice farming is practiced throughout the year with heavy insecticide use for crop protection. Due to that the farms are irrigated, breeding sites are present throughout the year and we therefore assumed that the larvae collected in the study period were representative of the population that could be found during other periods of the year. Larvae collected from multiple breeding sites were pooled together then re-distributed evenly in development trays containing tap water. Larvae were provided access to powdered TetraFin® fish food, and were reared to adults under insectary conditions of 25-28°C and 70-80% relative humidity at Centre Suisse de Recherches Scientifiques en Côte d’Ivoire (CSRS) located in Adiopodoumé. Non blood-fed adult female *An. gambiae s.l.*[12] at 1, 2, 3, 5 and 10 days post-emergence were tested using standard WHO susceptibility test kits [6] with insecticides belonging to the four classes used in public health, namely deltamethrin and permethrin (pyrethroids), DDT (organochlorine), malathion (organophosphate), and propoxur (carbamate). Non blood-fed female adults at 1–2 days post-emergence from the susceptible *An. gambiae* KISUMU strain were used as a control for each insecticide and each age group; only this age group was selected because this is the recommended age for WHO susceptibility assays [6]. Tests were performed following the standard protocol for adults at 25°C and relative humidity of 70-80%. Each complete bioassay was performed with five batches of 20–25 unfed females: four batches were exposed to impregnated filter papers and one non-exposed batch served as a control. The number of mosquitoes knocked down was recorded at 5 min intervals during the 1 hour exposure period and the mortality was determined 24 hours post-exposure. Bioassays were also performed with the susceptible reference KISUMU strain of *An. gambiae* maintained in the insectary at CSRS. Following the exposure, mosquitoes were supplied with 10% honey solution and kept overnight under laboratory conditions prior to noting the 24 hour mortality rates. WHO [6] criteria were used to indicate the susceptibility status. Results were compiled and analyzed using EpiInfo Version 6 [13] to test for any significant difference in mortality rates between the different age groups via Mentel-Haenszel Chi square test. The length of the exposure time at which 50% of the test population were knocked down (LT_50_) was determined using WinDL version 2.0 computer software (CIRADCA/MABIS, Montpellier, France), based on Finney (1971) log-probit model [14]. The resistance ratio (RR_50_) was determined relative to the KISIMU susceptible strain. This was obtained by dividing the LT_50_ of wild strain to the LT_50_ of the susceptible strain.

## Results

**Figure 1:**
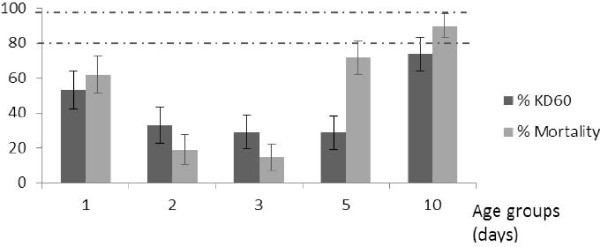
**Knockdown and mortality rates following exposure to deltamethrin of different ages of wild *An. gambiae *mosquitoes. **Vertical lines on top of the bars represent confidence intervals. Dotted lines represent upper (98%) and lower (80%) cut-offs for WHO classifications; values above the upper line indicate susceptibility, values within the two lines indicate possible resistance and values below the lower line indicate confirmed resistance (WHO, 1998).

**Figure 2:**
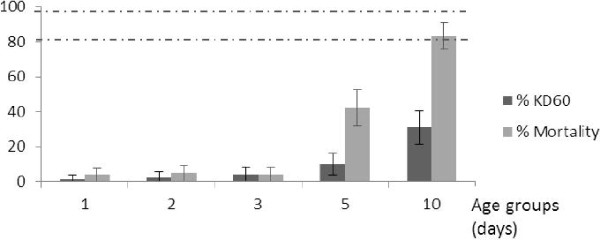
**Knockdown and mortality rates following exposure to permethrin of different ages of wild *An. gambiae *mosquitoes. **Vertical lines on top of the bars represent confidence intervals. Dotted lines represent upper (98%) and lower (80%) cut-offs for WHO classifications; values above the upper line indicate susceptibility, values within the two lines indicate possible resistance and values below the lower line indicate confirmed resistance (WHO, 1998).

For each insecticide and age group, no mortality was found in the control group hence validating the results from our assays. Results from another study held at the same time on the same field population revealed that all the individuals were *An. gambiae* s.s. from the M molecular form (Chouaibou et al., personal communication). The LT_50_ of the wild population of *An. gambiae s.l.* from Tiassalé, varied from 45.7 – 80.9 minutes for deltamethrin and 69.6 – 193.3 minutes for permethrin versus 15.1 and 14.6 minutes for the susceptible KISUMU strain for the same insecticides, respectively (Table 1). This indicated decreased susceptibility of the wild population to pyrethroids with a RR_50_ varying between 3.01 and 5.33 for deltamethrin and between 4.7 and 13.25 for permethrin. Mortality data also indicated decreased susceptibility to both deltamethrin and permethrin (Figure 1 and 2). A positive correlation was observed between KD and mortality for both deltamethrin (r = 0.55) and permethrin (r = 0.97). There was significant variation in both LT_50_ and mortality depending on the age of adults tested. For deltamethrin, LT_50_ was longest for 2 day olds with RR_50_ = 5.33 and shortest for the 10 days old individuals with the RR_50_ = 3.01. Mortality was also lowest for the 2 and 3 day old mosquitoes (19% and 15% respectively, P > 0.05) and highest for 10 day old mosquitoes (90%, P < 0.05). For permethrin, LT_50_ values were 164.0, 179.01 and 193.31 minutes respectively for 1, 2 and 3 day old mosquitoes, corresponding to a more than 10-fold higher LT_50_ that observed with the susceptible strain (RR_50_ = 11.24 - 13.25); LT_50_ was 69.64 minutes for 10 day old individuals corresponding to about 5-fold the LT_50_ of the susceptible colony (RR_50_ = 4.77); the values were intermediate for 5 day old mosquitoes (LT_50_ = 129.18 min; RR_50_ = 8.85). The lowest mortality rates were observed for 1 to 3 day old mosquitoes (<5%), intermediate for 5 day old (42%) and highest for 10 day old (83%) mosquitoes. When considering WHO susceptibility classifications [6], for deltamethrin and permethrin the population would have been classified as confirmed resistant if adults of less than 10 days old were used in assays, and possibly resistant if 10 day old mosquitoes were used.

**Table 1:** **Time required for 50% of the population to be knocked down (LT**_**50**_**) and resistance ratio relative to the susceptible strain (RR**_**50**_**) of different age groups of wild *An. gambiae *mosquitoes from Tiassalé following exposure to five insecticides**

**Insecticide**	**Age (days)**	**LT**_**50**_	**95% confidence interval**	**RR**_**50**_
Deltamethrin	*Kisumu (1–2)	15.18	(14.45-15-88)	1
	1	62.28	(57.46-71.03)	4.1
	2	80.93	(69.37-102.37)	5.33
	3	73.23	(65.20-86.94)	4.82
	5	72.7	(65.42-85.32)	4.79
	10	45.67	(43.84-47-90)	3.01
Permethrin	Kisumu (1–2)	14.59	(13.96-15.20)	1
	1	164.04	(105.91-497.81)	11.24
	2	179.01	(109.02-743.01)	12.27
	3	193.31	(112.62-951.82)	13.25
	5	129.18	(94.19-292.32)	8.85
	10	69.64	(63.50-79.50)	4.77
DDT	Kisumu (1–2)	21.37	(20.70-21.03)	1
	1	+∞	na	na
	2	+∞	na	na
	3	+∞	na	na
	5	201.2	(115.24-1129.87)	9.42
	10	+∞	na	na
Malathion	Kisumu (1–2)	24.93	(24.11-25.73)	1
	1	61.79	(56.86-69.04)	2.48
	2	54.66	(51.22-59.37)	2.19
	3	63.76	(58.59-71.63)	2.56
	5	38.31	(35.99-41.13)	1.54
	10	48.77	(46.08-52.24)	1.96
Propoxur	Kisumu (1–2)	27.09	(26.06-28.09)	1
	1	+∞	na	na
	2	+∞	na	na
	3	+∞	na	na
	5	64.61	(56.97-78.75)	2.39
	10	74.61	(67.48-89.31)	2.75

*Reference susceptible *An. gambiae s.s. *strain aged 1 to days old; na: not available; +∞: infinity.

**Figure 3:**
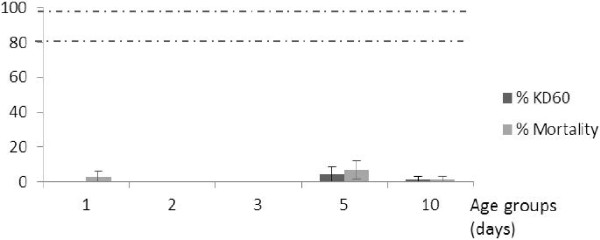
**Knockdown and mortality rates following exposure to DDT of different ages of wild *An. gambiae *mosquitoes. **Vertical lines on top of the bars represent confidence intervals. Dotted lines represent upper (98%) and lower (80%) cut-offs for WHO classifications; values above the upper line indicate susceptibility, values within the two lines indicate possible resistance and values below the lower line indicate confirmed resistance (WHO, 1998).

No KD effect was observed with DDT, except for the 5 day old individuals that had an associated RR_50_ of 9.42 (Table 1). According to WHO criteria, all the six age groups were classified as confirmed resistant as mortality was less than 7% or even nil (Figure 3), suggesting a very strong level of resistance of the Tiassalé population to DDT that was not affected by age.

**Figure 4:**
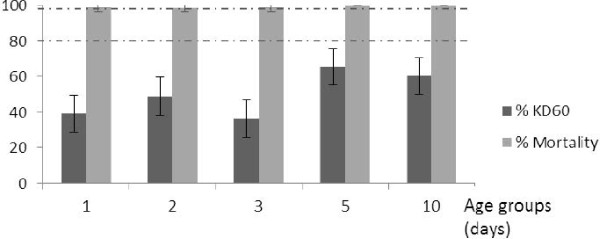
**Knockdown and mortality rates following exposure to malathion of different ages of wild *An. gambiae *mosquitoes. **Vertical lines on top of the bars represent confidence intervals. Dotted lines represent upper (98%) and lower (80%) cut-offs for WHO classifications; values above the upper line indicate susceptibility, values within the two lines indicate possible resistance and values below the lower line indicate confirmed resistance (WHO, 1998).

High mortality following exposure to the organophosphate malathion was observed for all age groups except the 5 day old mosquitoes, for which mortality was 93.6% (Figure 4). This shows possible resistance based on 5 day old mosquitoes and susceptibility for all other age groups tested. However, there was extended LT_50_ relative to the susceptible reference strain, with an RR_50_ of 1.54 for 5 day old mosquitoes (Table 1).

**Figure 5:**
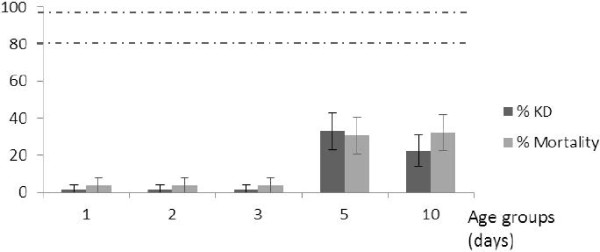
**Knockdown and mortality rates following exposure to propoxur of different ages of wild *An. gambiae *mosquitoes. **Vertical lines on top of the bars represent confidence intervals. Dotted lines represent upper (98%) and lower (80%) cut-offs for WHO classifications; values above the upper line indicate susceptibility, values within the two lines indicate possible resistance and values below the lower line indicate confirmed resistance (WHO, 1998).

Following exposure to propoxur (Figure 5), no KD effect was observed for 1, 2 and 3 day old individuals. A very low level of mortality was seen for 1, 2 and 3 day old mosquitoes (< 4%) while mortality was high for 5 to 10 day old mosquitoes (30%; P < 0.01). A positive correlation was observed between KD and mortality with propoxur over the different age groups (r = 0.90). This population would have been classified as confirmed resistant regardless of the age class used in WHO tests.

## Discussion

The impact of adult mosquito age on KD effect and mortality following exposure to various insecticides in WHO susceptibility tests, and also the resulting susceptibility status according to current WHO criteria [6], was assessed for a wild *An. gambiae* strain from Côte d’Ivoire.

Age-related variations in susceptibility to a particular insecticide were consistent for both knockdown and mortality rates. For the pyrethroids (i.e. deltamethrin and permethrin), a general increase in KD effect with mosquito age was observed, though this was non-linear for deltamethrin. A similar observation was made by Hodjati et al., [10], who showed a reduction in mean KD times of 10 day old adult *An. stephensi* and *An. gambiae*, relative to newly emerged mosquitoes. Another study has indicated that 14 day old mosquitoes were more rapidly knocked down than three day old mosquitoes, regardless of the strain or species [11]. For mortality, as also shown by others [15,16], older mosquitoes were more susceptible to insecticides. The impact of age was particularly marked for permethrin and propoxur and to a lesser degree with deltamethrin, with younger individuals being the least susceptible. For DDT, the influence of age on the mortality could not be assessed as all the age groups exhibited high resistance. The converse situation applied to malathion, in which all the age groups assessed were almost fully susceptible. Nevertheless, susceptibility to malathion has been shown to vary by mosquito age for *An. stephensi* from Pakistan [17]. Despite the consistently high mortality following exposure to malathion, observed differences in RR_50_ by mosquito age suggest that this is a sensitive indicator of reductions in susceptibility to malathion relative to a susceptible strain.

The long term and extensive use of DDT over the last few decades in Côte d’Ivoire [18] would have led to a strong selective pressure on exposed mosquito populations and could explain the high resistance to DDT observed in the study population. This observation highlights a concern that although pyrethroids are still effective, extensive and exclusive use of these compounds could lead to a situation where even the oldest individuals are no longer susceptible. This may have serious consequences for insecticide-based vector control programmes.

Cross-resistance between DDT and pyrethroids has been extensively demonstrated to be associated with *kdr* point mutations in the common target site of these insecticides [1,3,19]. In the current study, the large disparity in mortality rates after exposure to DDT versus the two pyrethroids suggests that *kdr* alone is likely to confer greater resistance to DDT compared to pyrethroids (at least in this population) and/ or several different mechanisms may confer resistance in the studied mosquito population. This was confirmed by the detection in this population of the *kdr* mutation at very high allelic frequency (94.5%) via molecular assays as well as indications of the involvement of up-regulated non-specific esterases (NSE) and mixed function oxidases (MFO) demonstrated by synergist tests via CDC bottle bioassays (Chouaibou et al., personal communication). Furthermore, insensitive acetylcholinesterase (*ace-1*^*R*^) due to the G119S mutation responsible for resistance to carbamates and organophosphates has also been described in this population with an allelic frequency of 50% [20]. In addition to the likely involvement of NSE, MFO, *ace-1*^*R*^and *kdr* mutations, studies using other *An. gambiae* populations have shown that a number of other mechanisms, such as oxidative stress reduction, can be involved in insecticide resistance [21-25]. The involvement of GST’s also merits further investigation as this could contribute to the higher level of DDT resistance relative to the pyrethroid resistance observed. Co-existence of all of these mechanisms would result in a large fitness cost, which may ultimately weaken older mosquitoes and render them more vulnerable to insecticides. Thus, there might be a trade-off between energy used for defense against insecticides and the onset of senescence, however, this requires further investigations. Moreover, changes in mosquito physiology that is not specifically associated with insecticides but that occurs with senescence such as an increase in the rate of cuticle permeability or a decrease in the rate of xenobiotic excretion, could also lead to an increase in susceptibility to insecticides. Increases in insecticide susceptibility in mosquitoes with aging may therefore explain observations relating to a lack of impact of observed phenotypic resistance on malaria epidemiological outcomes in areas with high insecticide treated net coverage [26,27], since the infective stage of the malaria parasites are more often harbored by the older *Anopheles spp.* that are presumably the most susceptible to insecticides. This hypothesis was recently confirmed by Jones et al. [28], who showed that as malaria vectors aged they became increasingly susceptible to pyrethroid based long-lasting insecticidal nets. Results from the current study showed that 1 to 3 days old adult females from the test population from Tiassalé, were the most resistant, thereby supporting the WHO recommendation to conduct assays on mosquitoes at 24–48 hours post-emergence [6]. The use of these younger and less susceptible mosquitoes in assays for phenotypic resistance should allow detection of resistance when the highest rate of the resistant phenotype is present.

## Conclusion

Using the WHO recommended age for insecticide susceptibility testing will enable detection of resistance where the highest rate of the resistant phenotype is present. However, the operational impact of that resistance may be less than often assumed, as the older mosquitoes that transmit malaria may still be affected by insecticide-based vector control. The current study highlighted the need adhere to standard procedures described in the WHO susceptibility test in order to generate accurate and exploitable data to more accurately inform choice of appropriate insecticidal vector control interventions.

## Competing interests

HPJ, GB, TK, JC, and LN are employed by Vestergaard Frandsen.

## Authors’ contributions

MC designed, implemented and coordinated the study, analyzed and interpreted data and drafted the manuscript. HPJ and GB conceived the study and revised the manuscript. TK and BB participated in the study design and conception and critically revised the manuscript. JC, LN and NK carried out field sampling and testing. All authors read and approved the final manuscript.

## Pre-publication history

The pre-publication history for this paper can be accessed here:

http://www.biomedcentral.com/1471-2334/12/214/prepub

## References

[B1] Martinez-Torres D Chandre F Williamson MS Darriet F Bergé JB Devonshire AL Guillet P Pasteur N Pauron D Molecular characterisation of pyrethroid knockdown resistance (kdr) in major malaria vector Anopheles gambiae s.s Insect Mol Biol 1998 7 179 184 10.1046/j.1365-2583.1998.72062.x 9535162

[B2] Weill M Lutfalla G Mogensen K Chandre F Berthomieu A Berticat C Pasteur N Phillipe N Fort P Raymond M Comparative genomics: Insecticide resistance in mosquito vectors Nature 2003 423 136 137 12736674 10.1038/423136b

[B3] Ranson H Jensen B Vulule JM Xang X Hemingway J Collins FH Identification of a novel mutation in the sodium voltage-gated sodium channel gene of Anopheles gambiae associated with resistance to pyrethroids insecticides Insect Mol Biol 2000 9 491 497 10.1046/j.1365-2583.2000.00209.x 11029667

[B4] Vontas JG Small GJ Hemingway J Glutathione-S-transferase as antioxidant defense agents confer pyrethroid resistance in Nilaparvata lugens Biochem J 2001 357 65 72 10.1042/0264-6021:3570065 11415437 PMC1221929

[B5] Vulule JM Beach RF Atieli FK McAllister JC Brogdon WG Roberts JM Mwangi RW Hawley WA Elevated oxidase and esterase levels associated with permethrin tolerence in Anopheles gambiae from Kenyan villages using permethrin-impregnated nets Med Vet Entomol 1999 13 239 244 10.1046/j.1365-2915.1999.00177.x 10514048

[B6] WHO Test procedures for insecticide resistance monitoring in malaria vectors, bioefficacy and persistence of insecticides on treated surfaces 12

[B7] Chouaïbou M Etang J Brévault T Nwane P Kérah Hinzoumbé C Mimpfoundi R Simard F The dynamics of insecticide resistance in the malaria vector Anopheles gambiae s.l. from an area of extensive cotton cultivation in Northern Cameroon Trop Med Int Health 2008 13 1 11 18248566 10.1111/j.1365-3156.2008.02025.x

[B8] Himeidan YE Hong Chen Chandre F Donnelly MJ Guiyan Y Permethrin and DDT Resistance in the Malaria Vector Anopheles arabiensis from Eastern Sudan AmJTrop Med Hyg 2007 77 1066 1068 18165523

[B9] Skovmand O Bonnet J Pigeon O Corbel V Median knock-down time as a new method for evaluating insecticide-treated textiles for mosquito control Malar J 2008 7 114 10.1186/1475-2875-7-114 18582393 PMC2459197

[B10] Hodjati MH Curtis CF Evaluation of the effect of mosquito age and prior exposure to insecticide on pyrethroid tolerance in Anopheles mosquitoes (Diptera: Culicidae) Bull Entomol Res 1999 89 329 337

[B11] Rajatileka S Burhani J Ranson H Mosquito age and susceptibility to insecticides Trans R Soc Trop Med Hyg 2011 105 247 253 10.1016/j.trstmh.2011.01.009 21353689

[B12] Gillies MT De Meillon B The anophelinae of Africa South of the Sahara Publication of the South African Institute for Medical Research 1968 54 343 p

[B13] Dean AG Dean JA Coulombier D Brendel KA Smith DC Burton AH Dicker RC Sullivan K Fagan RF Arner TG Epi Info Version 6: A Word Processing, Database, and Statistics Program for Epidemiology on Microcomputers 1994 Atlanta, GA: Center of Diseases Control and Prevention

[B14] Finney DJ Probit Analysis 1971 Cambridge, UK: Cambridge University Press

[B15] Glunt KD Thomas MB Read AF The effects of age, exposure history andmalaria infection on the susceptibility of Anopheles mosquitoes to low concentrations of Pyrethroid PLoS One 2011 6 e24968 10.1371/journal.pone.0024968 21966392 PMC3178580

[B16] Hunt RH Brooke BD Pillay C Koekemoer LL Coetzee M Laboratory selection for and characteristics of pyrethroid resistance in the malaria vector Anopheles funestus Med Vet Ent 2005 19 271 275 10.1111/j.1365-2915.2005.00574.x 16134975

[B17] Rowland M Hemingway J Changes in malathion resistance with age in Anopheles stephensi from Pakistan Pest Biochem Physiol 1987 28 239 47 10.1016/0048-3575(87)90022-8

[B18] Musawenkosi LH Sharp B Lengeler C Historical review of malarial control in southern African with emphasis on the use of indoor residual house-spraying Trop Med Int Health 2004 9 846 856 10.1111/j.1365-3156.2004.01263.x 15303988

[B19] Chandre F Darriet F Manguin S Brengues C Carnevale P Guillet P Pyrethroid cross resistance spectrum among populations of Anopheles gambiae s.s. from Côte d’Ivoire J Am Mosq Control Assoc 1999 15 53 59 10342269

[B20] Alou L Koffi A Adja M Tia M Kouassi P Koné M Chandre F Distribution of ace-1R and resistance to carbamates and organophosphates in Anopheles gambiae s.s. populations from Côte d'Ivoire Malar J 2010 9 167 10.1186/1475-2875-9-167 20553593 PMC2908637

[B21] David JP Strode C Vontas J Nikou D Vaughan A Pignatelli PM Louis C Hemingway J Ranson H The Anopheles gambiae detoxification chip: a highly specific microarray to study metabolic-based insecticide resistance in malaria vectors Proc Natl Acad Sci USA 2005 102 4080 4084 10.1073/pnas.0409348102 15753317 PMC554807

[B22] Ding Y Ortelli F Rossiter LC Hemingway J Ranson H The Anopheles gambiae glutathione transferase supergene family: annotation, phylogeny and expression profiles BMC Genomics 2003 4 35 10.1186/1471-2164-4-35 12914673 PMC194574

[B23] Müller P Chouaïbou M Pignatelli P Etang J Walker ED Donnelly JM Simard F Ranson H Tolerance to pyrethroids in Anopheles arabiensis is associated with elevated levels of antioxidant genes and correlates with agricultural use of insecticides Mol Ecol 2008 17 1145 1155 18179425 10.1111/j.1365-294X.2007.03617.x

[B24] Vontas J Blass C Koutsos AC David JP Kafatos FC Louis C Hemingway J Christophides GK Ranson H Gene expression in insecticide resistant and susceptible Anopheles gambiae strains constitutively or after insecticide exposure Insect Mol Biol 2005 14 509 521 10.1111/j.1365-2583.2005.00582.x 16164607

[B25] Pedra JHF McIntyre LM Scharf ME Pittendrigh BR Genome-wide transcription profile of field- and laboratory-selected dichlorodiphenyltrichloroethane (DDT)- resistant Drosophila Proc Natl Acad Sci USA 2004 101 7034 39 10.1073/pnas.0400580101 15118106 PMC406461

[B26] Henry MC Assi SB Rogier C Dossou-Yovo Chandre F Guillet P Carnevale P Protective efficacy of lambda-cyhalothrin treated nets in Anopheles gambiae pyrethroid resistant areas of Côte d’Ivoire AmJTrop Med Hyg 2005 73 859 864 16282294

[B27] Henry MC Doannio JM Darriet F Nzeyimana I Carnevale P Efficacité des moustiquaires pré-imprégnées Olyset Net® en zone de résistance des vecteurs aux pyréthrinoïdes Médecine tropicale 1999 59 355 7 10816747

[B28] Jones CM Sanou A Guelbeogo WM Sagnon N Johnson PC Ranson H Aging partially restores the efficacy of malaria vector control in insecticide-resistant populations of Anopheles gambiae s.l. from Burkina Faso Malar J 2012 11 24 10.1186/1475-2875-11-24 22269002 PMC3312828

